# A New Detection Method Defining the Aerobic Threshold for Endurance Exercise and Training Prescription Based on Fractal Correlation Properties of Heart Rate Variability

**DOI:** 10.3389/fphys.2020.596567

**Published:** 2021-01-15

**Authors:** Bruce Rogers, David Giles, Nick Draper, Olaf Hoos, Thomas Gronwald

**Affiliations:** ^1^College of Medicine, University of Central Florida, Orlando, FL, United States; ^2^Lattice Training Ltd., Chesterfield, United Kingdom; ^3^School of Health Sciences, College of Education, Health and Human Development, University of Canterbury, Christchurch, New Zealand; ^4^Center for Sports and Physical Education, Julius Maximilians University of Wuerzburg, Wuerzburg, Germany; ^5^Department of Performance, Neuroscience, Therapy and Health, Faculty of Health Sciences, MSH Medical School Hamburg, University of Applied Sciences and Medical University, Hamburg, Germany

**Keywords:** detrended fluctuation analysis, ventilatory threshold, aerobic threshold, intensity distribution, intensity zones, endurance exercise, endurance training, polarized training

## Abstract

The short-term scaling exponent alpha1 of detrended fluctuation analysis (DFA a1), a nonlinear index of heart rate variability (HRV) based on fractal correlation properties, has been shown to steadily change with increasing exercise intensity. To date, no study has specifically examined using the behavior of this index as a method for defining a low intensity exercise zone. The aim of this report is to compare both oxygen intake (VO_2_) and heart rate (HR) reached at the first ventilatory threshold (VT1), a well-established delimiter of low intensity exercise, to those derived from a predefined DFA a1 transitional value. Gas exchange and HRV data were obtained from 15 participants during an incremental treadmill run. Comparison of both VO_2_ and HR reached at VT1 defined by gas exchange (VT1 GAS) was made to those parameters derived from analysis of DFA a1 reaching a value of 0.75 (HRVT). Based on Bland Altman analysis, linear regression, intraclass correlation (ICC) and *t* testing, there was strong agreement between VT1 GAS and HRVT as measured by both HR and VO_2_. Mean VT1 GAS was reached at 39.8 ml/kg/min with a HR of 152 bpm compared to mean HRVT which was reached at 40.1 ml/kg/min with a HR of 154 bpm. Strong linear relationships were seen between test modalities, with Pearson’s *r* values of 0.99 (*p* < 0.001) and.97 (*p* < 0.001) for VO_2_ and HR comparisons, respectively. Intraclass correlation between VT1 GAS and HRVT was 0.99 for VO_2_ and 0.96 for HR. In addition, comparison of VT1 GAS and HRVT showed no differences by *t* testing, also supporting the method validity. In conclusion, it appears that reaching a DFA a1 value of 0.75 on an incremental treadmill test is closely associated with crossing the first ventilatory threshold. As training intensity below the first ventilatory threshold is felt to have great importance for endurance sport, utilization of DFA a1 activity may provide guidance for a valid low training zone.

## Introduction

Training zone identification is part of the foundation for exercise intensity distribution study and implementation (Stöggl and Sperlich, 2019. Traditionally, the upper limit of the low intensity range (zone 1 in a 3 zone model) for intensity distribution for endurance exercise and training prescription has been represented by the first ventilatory (VT1) or lactate threshold (LT1) (Seiler and Kjerland, 2006; Esteve-Lanao et al., 2007; Mann et al., 2013; Pallarés et al., 2016). Although there may be different schools of thought on what type of distribution is “optimal” (polarized vs. pyramidal or threshold) both models are defined by having the major portion of training in zone 1. In addition, several training approaches for endurance athletes recommend spending large amounts of exercise time in a low intensity zone (Muñoz et al., 2014; Stöggl and Sperlich, 2014, 2019; Bourgois et al., 2019; Casado et al., 2019). Gold standard methods to obtain VT1 or LT1 revolve around either formal gas exchange testing or invasive blood lactate sampling. These procedures can be costly, require special test equipment, trained operators, ongoing calibration and verification. Even if these methods are utilized, there is disagreement on their accuracy as both visual (Yeh et al., 1983; Meyer et al., 1996) and automated (Ekkekakis et al., 2008) gas exchange analysis can be subject to substantial error. In addition, LT1 assessment can vary depending on the chosen concept of determination (Newell et al., 2007; Faude et al., 2009; Jamnick et al., 2018). Training guided by erroneous intensity targets could lead to potential adverse consequences such as prolonged cardiac parasympathetic recovery (Seiler and Kjerland, 2006; Stanley et al., 2013), central and muscular fatigue (Noakes et al., 2005; Venhorst et al., 2018), glycogen depletion (Beneke et al., 2011), and gastrointestinal barrier disruption (van Wijck et al., 2012). In view of the difficulties involved in gas exchange analysis, lactate test availability, invasiveness, and accuracy, a search for alternate methods of identifying the limits of low intensity exercise seem worthwhile.

Cardiac interbeat interval variation, commonly referred to as heart rate variability (HRV), has been extensively studied in both resting states (Shaffer and Ginsberg, 2017) as well as during dynamic exercise (Hottenrott and Hoos, 2017; Michael et al., 2017). Certain HRV indexes have been observed to change as exercise intensity rises, potentially providing information regarding an individual’s physiologic status (Tulppo et al., 1996; Casties et al., 2006; Sandercock and Brodie, 2006; Karapetian et al., 2008; Michael et al., 2017; Gronwald et al., 2018, 2019a,b, 2019c). It has also been shown that several of the examined HRV indexes also change during lower intensities (Tulppo et al., 1996; Sandercock and Brodie, 2006; Karapetian et al., 2008; Botek et al., 2010; Michael et al., 2017) making them potentially suitable for zone 1 delineation. However, despite some initial interest, widespread usage for the specific purpose of low intensity training limitation has not occurred. Frequency-domain parameters such as high frequency (HF) power have been noted to be unreliable in a sizable fraction of individuals with up to 20% of subjects not having identifiable breakpoints (Cottin et al., 2007). Time domain measures such as SDNN were found to closely relate with VT1 but little follow-up or verification has been done (Karapetian et al., 2008). The SD1 is another index that has been examined during exercise. It is based on a Poincare plot of each RR interval graphed against the preceding interval and is related to short term trends in RR patterns often assigned to nonlinear indexes (Shaffer and Ginsberg, 2017), although it is mathematically equivalent to another time domain index (Ciccone et al., 2017). While showing potential as a low intensity marker in some earlier studies (Tulppo et al., 1996) other evidence indicates that SD1 was already suppressed in young athletes at the first tested work rate of 60% VO_2MAX_ making it less useful for zone 1 delineation (Blasco-Lafarga et al., 2017).

One nonlinear index, the short-term scaling exponent alpha1 based on Detrended Fluctuation Analysis (DFA a1), has generated interest as both an indicator of autonomic nervous system regulation as well as an overall marker of organismic demands Gronwald and Hoos, 2020). Originally, Peng et al. (1995) developed this method to measure scale-invariant behavior; this involved the evaluation of trends of all sizes in the presence or absence of fractal correlation properties in a heart rate (HR) time series (Yeh et al., 2010). Thus, the DFA method allows for the quantification of the degree of correlation and fractal scale of a HRV signal resulting in dimensionless measures. The short-term scaling exponent DFA a1 is based on the fractal dynamics (self-similarity) of the cardiac beat-to-beat pattern and provides insights into correlation properties of HR time series caused by physiological processes (Peng et al., 1995). DFA a1 values indicate time series correlation properties with approximately 1.5 indicating a strongly correlated pattern and ≤0.5 for anti-correlated pattern with random behavior; approximately 1.0 signifies a mix of uncorrelated and maximally correlated signal components (represents a balance between complete unpredictability (randomness) and predictability (strong correlations), also associated with fractal (self-similar) behavior (Platisa and Gal, 2008). Larger values of DFA a1 represent a smoother time series and smaller values of DFA a1 represent coarser ones (Peng et al., 1995; Goldberger et al., 2002). Within this framework, DFA a1 has been shown to decline as work rate rises, starting from strongly correlated patterns (value of 1.5) at rates well below the first ventilatory threshold (VT1), transitioning (values of 1.0–0.5) through values representing uncorrelated, less complex white noise behavior at moderate to high work rates, then finally showing anti-correlated behavior at the highest intensities (values of <0.5) (Gronwald et al., 2019c; Gronwald and Hoos, 2020). Given this relationship, there may be an opportunity to assist athletes in delineating intensity training zones by observing the change in DFA a1 with increasing exercise intensity (Gronwald et al., 2020; Rogers, 2020).

The purpose of this report is to validate a predefined DFA a1 value of 0.75 with the exercise intensity at VT1 obtained during an incremental treadmill run to exhaustion. This is to be done by a direct comparison of the VT1 intensity based on both absolute VO_2_ and HR obtained during gas exchange with the same measures derived from analysis of DFA a1 behavior. If it can be shown that a predefined “boundary” value in the DFA a1 index occurs near the VT1, this could establish a basis for further research exploiting a non-linear autonomic nervous system related marker in prospective exercise and training intensity distribution.

## Materials and Methods

### Participants

Seventeen male volunteers aged 19–52, without previous medical history, current medications or physical issues were tested. A background questionnaire regarding medical history was reviewed along with information of the potential testing risks then institutionally approved consent was given. Approval for the study was granted by the University of Derby, United Kingdom (LSREC_1415_02) and conformed to the principles of the Declaration of Helsinki. Participants did not consume caffeine, alcohol or any stimulant for the 24 h before testing. Background data for each subject included, age, body weight, and training volume in hours per week (Table 1). All testing was done in the afternoon and at least 3 h post meal. No exercise was performed the day prior to the test. Two participants with a high degree of cardiac ectopy (frequent atrial premature beats and atrial trigeminy) during testing were excluded from analysis.

**TABLE 1:** Demographic data of all included participants (*n* = 15) with training volume.

Subject number	Age (years)	BW (Kg)	HT (cm)	TV (h/wk)
1	19	82	182	3–6
2	19	82	176	3–6
3	20	82	190	3–6
4	23	77	180	>6
5	24	69	171	3–6
6	24	65	165	>6
7	24	76	186	3–6
8	25	78	171	>6
9	26	69	169	>6
10	30	92	189	1–3
11	30	73	175	>6
12	32	65	161	1–3
13	36	75	182	>6
14	50	94	178	3–6
15	52	71	171	1–3
Mean (SD)	29 (±10)	77 (±9)	176 (±9)	-

*BW, Body weight; TV, Training volume. Mean (±standard deviation, SD) in last row.*

### Exercise Protocol

Participants performed an incremental VO_2MAX_ test on a motorized treadmill (Woodway, Birmingham, United Kingdom). The treadmill was set for the Bruce protocol with increases in speed and inclination from 2.7 km/h at ten percent grade, increasing by 1.3 km/h and two percent grade every 3 min until volitional exhaustion. A fan was used for cooling.

### Gas Exchange Testing and Calculation of the First Ventilatory Threshold

**FIGURE 1:**
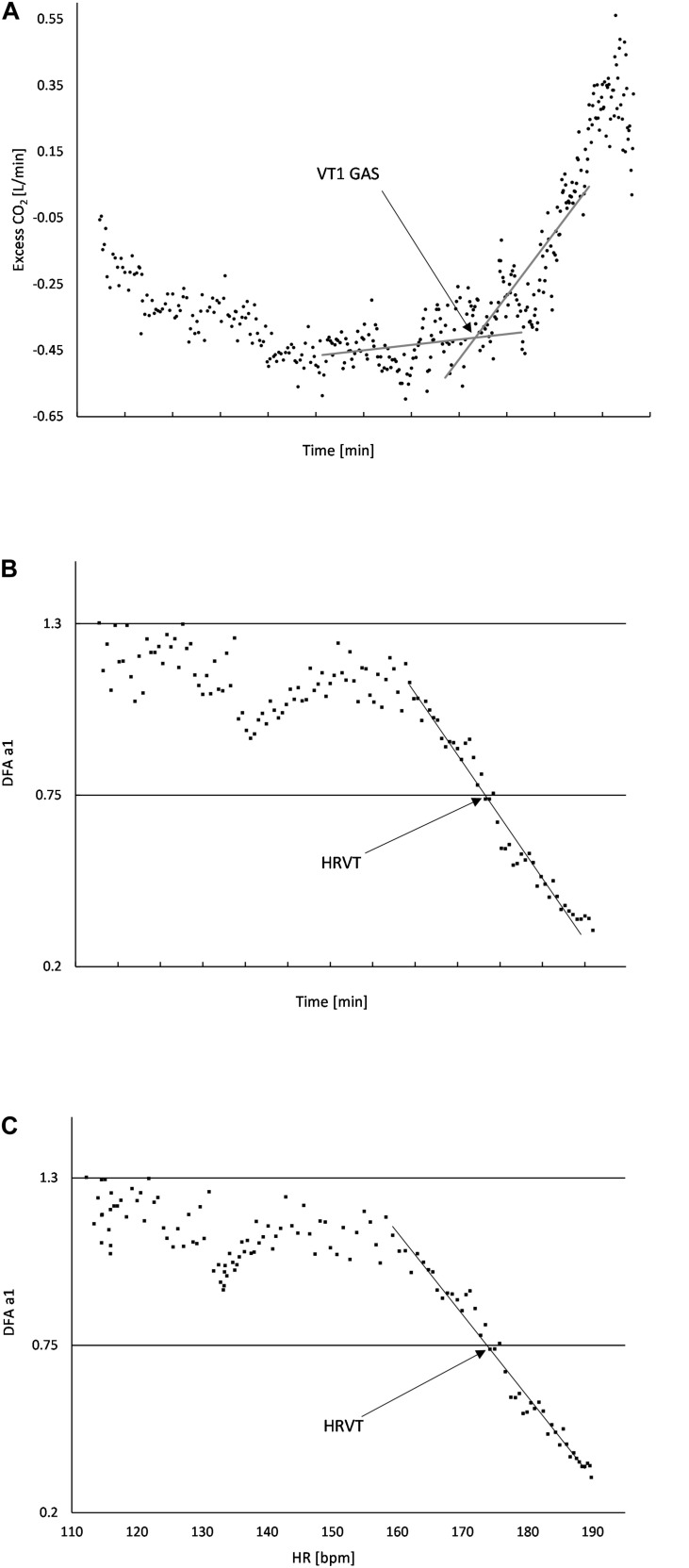
**(A)** Usage of “Excess CO_2_” technique to determine VT1 GAS. The intersection of the baseline and first rise in excess CO_2_ corresponds to the time when VT1 occurs; **(B)** DFA a1 plotted over time, the area of linear drop of DFA a1 from about 1.0 to 0.5 is used to determine the VO_2_ (from the VO_2_ vs. time relation) at HRVT; **(C)** DFA a1 plotted against HR, the area of linear drop of DFA a1 from 1.0 to 0.5 is used to determine the HR at HRVT. All data taken from Subject #2.

Gas exchange kinetics were recorded continuously using a breath-to-breath metabolic cart (Metalyzer 3B; Cortex Biophysik GmbH Germany). In addition, a Polar H7 (Polar Electro Oy, Kempele, Finland) was wirelessly paired to the Metalyzer cart for the purpose of HR recording concurrent with gas exchange data. VO_2_, VCO_2_, PetO_2_, PetCO_2_, Ve/VO_2_, Ve/VCO_2_, and HR were imported into Microsoft Excel 365 for analysis. The native gas exchange analysis feature of the Metalyzer was not used due to the unreliability of many automated VT1 calculations (Ekkekakis et al., 2008). Graphing of the above parameters were done to derive VT1, VO_2MAX_, and VO_2_ vs. time. No averaging was done for either gas exchange parameters or HR. Inspection of the VO_2_ over time relationship was done to determine any significant plateau of the VO_2_ curve for estimation of VO_2MAX_ and VO_2_ linearity. If a significant plateau was found, compensation for calculating both VO_2MAX_ and the VO_2_ over time equation was done. To reduce the chance of failure to identify the VT1 by gas exchange (VT1 GAS) based on a single method, evaluation was done according to the triple detection method consisting of V slope, Ve/VO_2_, and excess CO_2_ from Gaskill et al. (2001) as well as the PetO_2_ nadir from Binder et al. (2008). Based on the quality and consistency of the plots, the excess CO_2_ method was chosen to be used for all participants and reviewed independently by two investigators (Figure 1A). VO_2_ was plotted over the elapsed time of the incremental test to produce a linear regression equation. VO_2_ at the time of VT1 was based on linear regression from the VO_2_ over time relationship.

### RR Measurements and Calculation of DFA a1 Derived Threshold

A 3-lead ECG (MP36; Biopac Systems Ltd.) with a sampling rate of 1,000 Hz was used to record the subject’s ECG/RR times series. Biopac filter settings were set to 0.05 Hz high-pass filter and 150 Hz low-pass filter. Electrodes were placed in the CM5 distribution after appropriate skin cleansing and shaving if necessary. Sample data from the MP36 was saved as .acq files. ECG files for each subject were imported into Kubios 3.3.2 (Biosignal Analysis and Medical Imaging Group, Department of Physics, University of Kuopio, Kuopio, Finland). Kubios preprocessing settings were at the default values including the RR detrending method which was kept at “Smoothn priors” (Lambda = 500, Tarvainen et al., 2014). For DFA a1 estimation, the root mean square fluctuation of the integrated and detrended data is measured in observation windows of different sizes. The data are then plotted against the size of the window on a log-log scale. The scaling exponent represents the slope of the line, which relates (log) fluctuation to (log) window size (Mendonca et al., 2010). DFA a1 window width was set to 4 ≤ *n* ≤ 16 beats.

For the detection of a HRV derived threshold, a DFA a1 value of 0.75 was chosen based on this being the midpoint between a fractal behavior of the HR time series of 1.0 (seen with very light exercise) and an uncorrelated value of 0.5 which represents white noise, random behavior (seen with high intensity exercise). A value of 0.75 has also been used as a cut-off value for survival curves and mortality rate assessment during resting conditions (Huikuri et al., 2000).

The following procedure was used to indicate at what level of running intensity (as VO_2_ or HR) the DFA a1 would cross a value of 0.75: DFA a1 was calculated from the incremental exercise test RR series using 2 min time windows with a recalculation every 5 s throughout the test. Two minute time windowing was chosen based on the reasoning of Chen et al. (2002). The rolling time window measurement was used to better delineate rapid changes in the DFA a1 index over the course of the test. Each DFA a1 value is based on the RR series 1 min pre and 1 min post the designated time stamp. For example, at a time of 10 min into the testing, the DFA a1 is calculated from the 2 min window starting from minute 9 and ending at minute 11 and labeled as the DFA a1 at 10 min. Based on a rolling time recalculation every 5 s, the next data point would occur at 10:05 min (start 9:05 min and end 11:05 min).

Plotting of DFA a1 vs. time was then performed. Inspection of the DFA a1 relationship with time generally showed a reverse sigmoidal curve with a stable area above 1.0 at low work rates, a rapid, near linear drop reaching below 0.5 at higher intensity, then flattening without major change. A linear regression was done on the subset of data consisting of the rapid near linear decline from values near 1.0 (correlated) to approximately 0.5 (uncorrelated). The time of DFA a1 reaching 0.75 was calculated based on the linear regression equation from that straight section (Figure 1B). The time of DFA a1 reaching 0.75 was then converted to VO_2_ using the VO_2_ vs. time relation, resulting in the VO_2_ at which DFA a1 equaled 0.75 (HRVT). A similar analysis was done for the HR reached at a DFA a1 of 0.75. First, ECG data from each 2 min rolling window was used to plot the average HR and DFA a1. The HR at which DFA a1 equaled 0.75 was found using the same technique as above, a linear regression through the rapid change section of DFA a1 values of 1.0 to below 0.5, with a subsequent equation for HR and DFA a1 (Figure 1C). Using a fixed variable of DFA a1 equals 0.75, the resulting HR was obtained. The HR at DFA a1 0.75 (based on ECG data) was then compared to the HR at VT1 GAS obtained from the metabolic cart data (based on the Polar H7).

Visual inspection of the entire test recording was done to determine sample quality, noise, arrhythmia, and missing beat artifact. As mentioned above, two participants with a high degree of atrial ectopy were excluded from analysis. The RR series of the included participants was then corrected by the Kubios “automatic method” and exported as text files for further analysis. Percent artifact reported refers those occurring during the linear regression segment (DFA a1 1.0 to near 0.5).

### Statistics

Statistical analysis was performed for the main variables, VO_2_ at VT1 derived from gas exchange testing, VO_2_ at DFA a1 0.75, HR at VT1 obtained from gas exchange testing and average HR at DFA a1 0.75. Standard statistical methods were used for the calculation of means and standard deviations (SD). Normal distribution of data was checked by Shapiro–Wilk’s test. The agreement against the Gold Standard VT1 GAS was assessed using intraclass correlation coefficient (ICC), linear regression, Pearson’s *r* correlation coefficient, standard error of estimate (SEE), coefficient of determination (R^2^) and Bland Altman plots with limits of agreement (Bland and Altman, 1999). The size of Pearson’s *r* correlations evaluated as follows; 0.3 ≤ *r* < 0.5 low; 0.6 ≤ *r* < 0.8 moderate and *r* ≥ 0.8 high (Chan, 2003). The paired t-test was used for comparison of VT1 GAS vs. HRVT for both VO_2_ and HR parameters. For all tests, the statistical significance was accepted as *p* ≤ 0.05. Cohen’s *d* was used to denote effect sizes (small effect = 0.2, medium effect = 0.5, large effect = 0.8; Cohen, 1988). Analysis was performed using Microsoft Excel 365 with Real Statistics Resource Pack software (Release 6.8).

## Results

### Gas Exchange Testing

Individual gas exchange results are presented in Table 2. Both the VO_2MAX_ as well as the percentage of VO_2MAX_ and HR at VT1 GAS varied considerably among participants. VO_2MAX_ ranged between 41 and 74 ml/kg/min. VT1 GAS was reached between 61 and 86% of the VO_2MAX_ and at HRs between 108 and 183 bpm.

**TABLE 2:** Comparison of VT1 GAS and HRVT with measures of VO_2_ and HR.

Subject number	VO_2MAX_	VT1 GAS VO_2_	VT1 GAS VO_2_	HRVT VO_2_	VT1 GAS HR	HRVT HR	Artifacts (%)
	(ml/kg/min)	(%_MAX_)	(ml/kg/min)	(ml/kg/min)	(bpm)	(bpm)	
1	58	77	45.2	46.1	167	170	0
2	57	75	42.8	45.1	169	175	0.5
3	47	70	32.7	31.6	178	175	0
4	71	86	61.2	61.2	155	156	0
5	64	68	43.6	43.0	143	137	0
6	54	74	40.1	38.1	165	163	0
7	47	76	35.8	37.2	164	171	2
8	54	70	37.8	37.9	137	135	0
9	72	69	49.3	49.2	183	184	0
10	46	61	27.7	29.2	108	122	0
11	74	65	48.1	46.4	151	148	3
12	49	66	32.0	33.3	154	160	1
13	57	66	37.6	39.4	154	159	1
14	41	70	28.6	28.8	139	136	0
15	54	64	34.5	35.5	118	122	1
Mean (SD)	56 (±10)	70 (±6)	39.8 (±8.9)	40.1 (±8.6)	152 (±21)	154 (±20)	0.6 (±0.9)

*VT1 GAS, first ventilatory threshold; HRVT, DFA a1 derived threshold; HR, Heart rate; VO_2MAX_, VT1 GAS percent of VO_2MAX_ and Artifacts percentage. Mean (±standard deviation, SD) in last row.*

### RR Interval Quality

The percentage of artifacts was calculated based on the Kubios automatic correction method for each subject’s test data. Since only a portion of the entire treadmill test was used for the linear interpolation of DFA a1, the artifact percentage listed refers to that section only. Artifact percentage for the linear plotted data series was between 0 and 3%, all consisting of atrial premature complexes (Table 2). There were no missed beats due to noise interference or loss of electrode contact. The two participants originally excluded from analysis had significant ectopy, leading to an uninterpretable DFA a1 pattern.

### Comparison of VT1 GAS vs. HRVT

**FIGURE 2:**
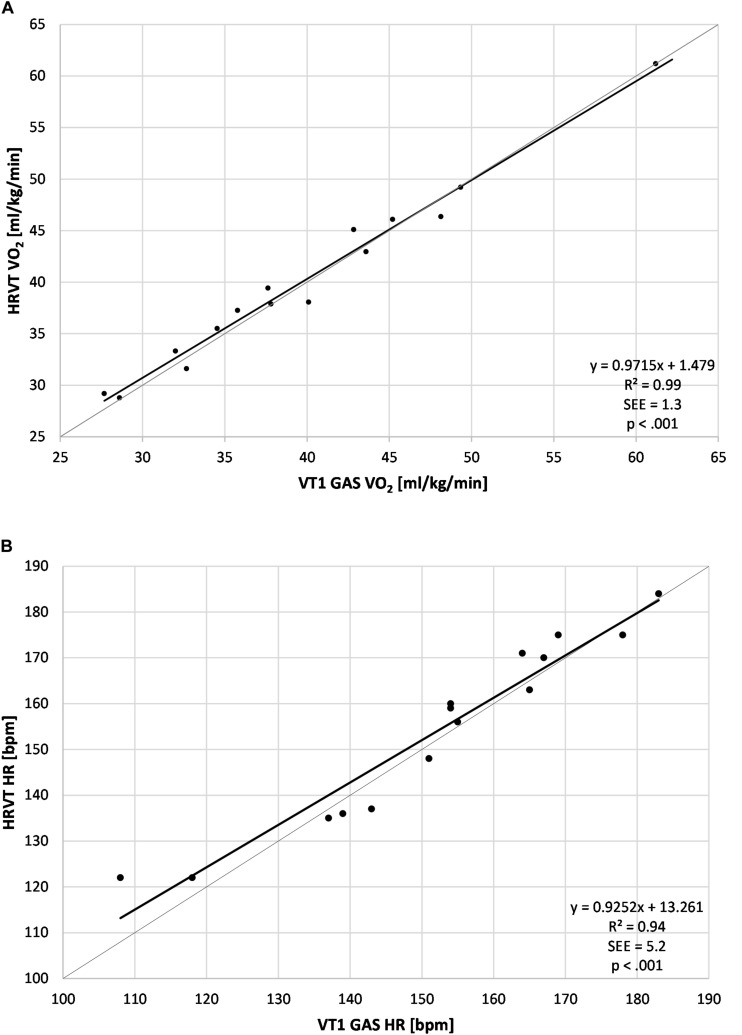
Regression plots for all subject data. **(A)** Values of VT1 GAS vs. HRVT for VO_2_; **(B)** Values of VT1 GAS vs. HRVT for HR. Bisection lines in light gray. SEE, standard error of estimate; R^2^, coefficient of determination.

**FIGURE 3:**
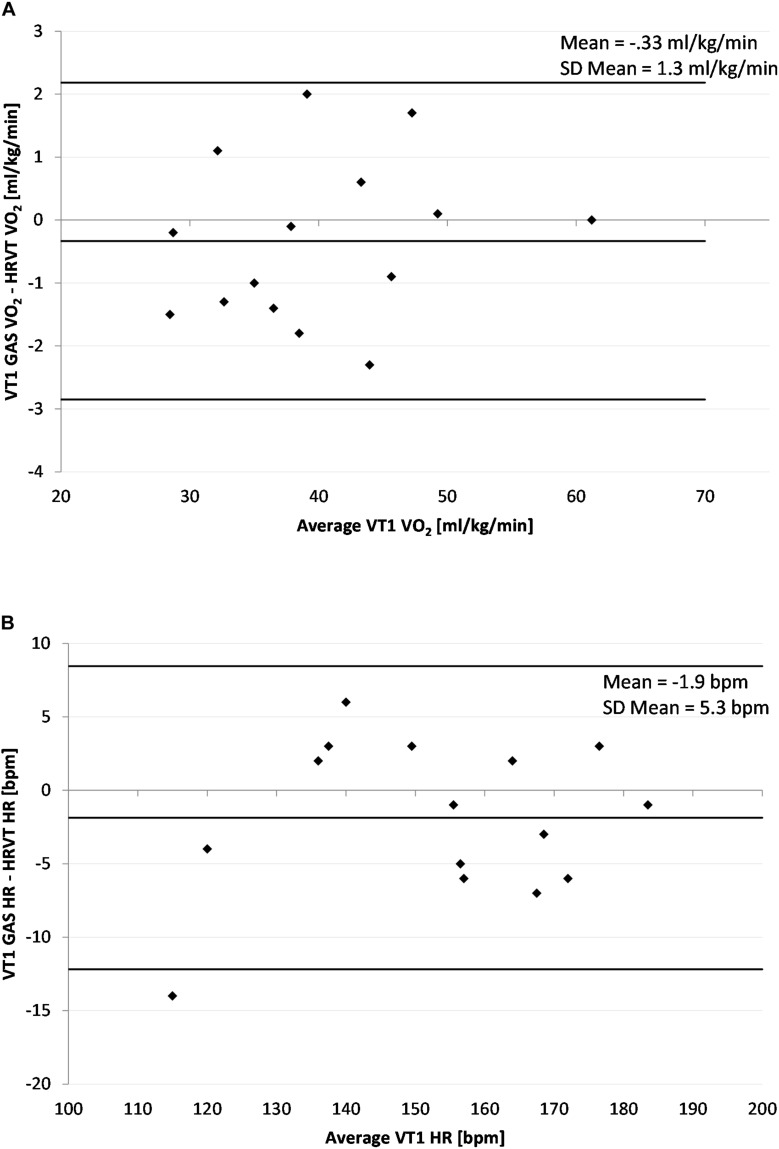
Bland Altman Plot of VT1 GAS vs. HRVT for all participants. **(A)** Values of VT1 GAS vs. HRVT for VO_2_; **(B)** Values of VT1 Gas vs. HRVT for HR. Center line in each plot represents the mean difference between each paired value, the top and bottom lines are 1.96 standard deviations from the mean difference.

The average VT1 GAS was 39.8 ml/kg/min (±8.9) compared to 40.1 ml/kg/min (±8.6) obtained by HRVT. The average HR at VT1 GAS was 152 bpm (±21) compared to 154 bpm (±20) obtained by HRVT. Strong linear relationships were seen between test modalities, with Pearson’s *r* values of 0.99 (*p* < 0.001) and.97 (*p* < 0.001) for VO_2_ and HR comparisons respectively (Figure 2). Intraclass correlation between VT1 GAS and HRVT was 0.99 for VO_2_ and 0.96 for HR. The comparison of VT1 GAS and HRVT showed no differences (VO_2_: *p* = 0.347, *d* = 0.030; HR: *p* = 0.191, *d* = 0.091). Bland Altman analysis for VT1 GAS vs. HRVT for VO_2_ (Figure 3) showed a mean difference of −0.33 ml/kg/min (±1.3) with upper and lower limits of 2.2 and −2.9 ml/kg/min. Bland Altman analysis for VT1 GAS vs. HRVT for HR (Figure 3) showed a mean difference of −1.9 bpm (±5) with upper and lower limits of 8 and −12 bpm.

## Discussion

This study explored whether values of the nonlinear HRV index, DFA a1, pass through a defined transitional zone at workloads near VT1 during an incremental treadmill test. Since many prior reports have shown DFA a1 to decline during incremental exercise (Hautala et al., 2003; Casties et al., 2006; Platisa et al., 2008; Karavirta et al., 2009; Blasco-Lafarga et al., 2017; Gronwald et al., 2019c), our result showing a similar occurrence is not unanticipated. However, none have attempted to directly examine the possibility that the DFA a1 index has a distinct value at the VT1 work rate. Since many of the prior studies looking at DFA a1 response to incremental exercise intensity have used cycling as the exercise modality, it is also reassuring to see analogous results with treadmill running, adding validity to the behavior of this index during other endurance exercise types. The inclusion of a wide range of subject ages, body weights and fitness abilities, lends strength to the application of our results to the general population and its application in different fields of physical exercise and training.

In a recent perspective review (Gronwald et al., 2020), identification of a low intensity exercise zone based on DFA a1 for the purposes of endurance exercise and training prescription was discussed. The mechanism underlying DFA a1 decline with exercise is felt to be related to autonomic balance and a complex interaction of the two main branches, namely parasympathetic withdrawal, sympathetic intensification as well as other factors (Gronwald and Hoos, 2020). Since VT1 is usually seen at a point of significant parasympathetic withdrawal (Tulppo et al., 1996; Sales et al., 2019), leveraging HRV parameters that reliably reflect this occurrence can be of use during endurance exercise and training. Our methodology to determine HRVT utilized the rapid decline of DFA a1 from 1.0 to below 0.5, seen during progressive exercise intensity. The results presented here appear to indicate that VT1 is reached at a midpoint between a fractal behavior of DFA a1 and a pattern of uncorrelated white noise with random behavior, corresponding to a DFA a1 of approximately 0.75. Bland Altman analysis with limits of agreement showed minimal difference between VT1 GAS and HRVT looking at either VO_2_ or HR measurements. Correlation coefficients and ICC were high for both VO_2_ and HR based comparisons.

Although the DFA a1 value of 0.75 was chosen theoretically, a brief review of prior investigation is supportive of this figure. In a study of young men performing a cycling ramp test, an average DFA a1 of 0.49 was associated with a lactate measurement of 2.49, indicating that LT1 had already been exceeded (Gronwald et al., 2019c). Other cycling ramp studies in men of different fitness levels seemed to indicate that DFA a1 crossed the value of 0.75 at about 73–78% of VO_2MAX_ (Hautala et al., 2003; Hottenrott and Hoos, 2017), within the approximate realm of VT1 for many individuals (Gaskill et al., 2001; Pallarés et al., 2016). An examination of the DFA a1 response to incremental cycling exercise in teenage males (Blasco-Lafarga et al., 2017) showed an approximate crossing of the 0.75 value at an average intensity near 65% of maximum, also near published ranges of VT1 (50–65% of VO_2MAX_) in that age group (Runacres et al., 2019). In the current study, there appeared to be little bias in the VO_2_ or HR associated with HRVT. Although there was some variability in VT1 GAS vs. HRVT parameters, for the most part, associations were similar to that of other comparisons of threshold approaches such as blood lactate or ventilatory parameters (Pallarés et al., 2016), or assessment of gas exchange techniques for VT1 determination (Gaskill et al., 2001). Several participants had relatively high heart rate at the VT1 (Azevedo et al., 2011) of which we do not have an explanation. A strength of this study is that of RR interval quality. Direct ECG visualization was done and a research grade device with a high sample rate was used. No missed or lost beats were seen, and the only artifact type present was APC aberrancy. In view of reports indicating substantial bias in nonlinear HRV indexes with artifact presence (Giles and Draper, 2018; Rincon Soler et al., 2018), a weakening of DFA a1 derived VT1 accuracy could occur with higher artifact occurrence.

A significant advantage of DFA a1 over other HRV indexes for the determination of a low intensity threshold revolves around the nature of testing. Other HRV metrics proposed to identify VT1 such as SDNN (Karapetian et al., 2008), HF power (Cottin et al., 2007) or SD1 (Tulppo et al., 1996) require testing into high intensity zones since they rely on curve interpretation that displays a demonstrable nadir. With DFA a1, once the VT1 boundary area is reached, little additional increase in exercise intensity should be required. The potential benefit of utilizing a fixed DFA a1 value as the VT1 delineation marker is especially attractive in populations unable or ill advised to enter high intensity regions. In addition, for athletes evaluating low intensity training limits, avoidance of exercise ramps to volitional failure may help avert undue stress in a polarized training model.

### Limitations and Future Direction

Given the issues with both availability and accuracy of gas exchange or blood lactate testing in determining VT1 for training zone purposes, an alternate modality that employs relatively simple wearable technology seems attractive. However, while DFA a1 monitoring may be a promising approach, several questions need to be addressed. Although this study was done with a wide range of subject age and fitness characteristics, no female participants were tested. If the DFA a1 index behavior is to be considered as a zone 1 delimiter for the general population, further investigation using female subjects is mandatory. Another area of concern is the transfer of the DFA a1 0.75 breakpoint obtained during incremental testing to that of one found during constant load exercise, including moderate length intervals (5 min). No data is available comparing DFA a1 behavior during an incremental ramp to constant load exercise (Gronwald and Hoos, 2020), making automatic transfer of zone boundaries unclear. Whether the index will remain stable for even longer exercise intervals (>60 min) performed below VT1 intensity is another open question as well as day to day repeatability. Another interesting subject to explore is the impact of athlete overtraining on DFA a1 behavior and VT1 prediction accuracy during exercise. Baumert et al. (2006) did show changes in DFA related scaling behavior after intense training, which may provide both a potential source of HRVT bias and an opportunity to screen for overtraining states. Although it seems that ramp protocol slope has minimal effect on the VT1 gas assessment (Weston et al., 2002; Boone and Bourgois, 2012), the analogous assumption needs to be shown in terms of HRVT thresholds. Another area for investigation is whether DFA a1 cut off values are equivalent between chest belt and research grade ECG recordings. Although in this study, the RR intervals were recorded with a research grade ECG device, it may be possible to reproduce similar results with chest belt ECG recordings. In that regard, two major questions need to be addressed. One is that of exercise associated missed beat artifact with possible faulty interpolation strategies by interpreting software, creating potential bias in the calculated DFA a1 values. As mentioned above, several reports have questioned the degree of bias of nonlinear HRV indexes if artifacts are present in the RR series (Giles and Draper, 2018; Rincon Soler et al., 2018; Stapelberg et al., 2018). Artifacts may be of different types such as missed beats or aberrancy. In the current report, no missed beats were seen, and only relatively rare atrial premature complexes were noted. However, two participants exhibited frequent APC aberrancy, had uninterpretable DFA a1 curves and were excluded from group analysis. Given the relative low numbers of participants, no definitive conclusion can be reached regarding artifact bias, but further investigation into effects of missed beats and aberrancy on the use of DFA a1 to delineate zone 1 transition is needed. Second is the question of DFA a1 value precision obtained by diverse monitoring devices possessing different sample rates and prepossessing strategies. Device sample rates have been shown to variably alter DFA a1 values at rest (Voss et al., 1996; Tapanainen et al., 1999; Singh et al., 2015) but may have more significant effects during exercise. Although no recent caffeine use was noted by history, we have no information on prior long term intake patterns which could affect autonomic balance on abrupt discontinuation (La Monica et al., 2018). Finally, it may be possible to answer many of these questions by “repurposing” prior work already done. For instance, a study by Boullosa et al. (2014) assessed the changes in DFA a1 before and after a typical incremental treadmill ramp to exhaustion. A look back at previously acquired RR recordings during the active ramp portion using the methods discussed here may be a way to rapidly acquire needed information about DFA a1 behavior during dynamic exercise.

### Conclusion

DFA a1, an index of fractal dynamics and correlations properties of the heart rate time series, was noted to decline during an incremental treadmill run test to exhaustion. The area of most rapid change of this index occurred near the first ventilatory threshold. The point of DFA a1 reaching a value of 0.75 during the incremental treadmill test was directly associated with the first ventilatory threshold as measured by heart rate and gas exchange VO_2_. As training intensity below the first ventilatory threshold is felt to have great importance for exercise and training prescription in endurance sport, utilization of DFA a1 activity may provide guidance for a valid low training zone boundary without the need for gas exchange or blood lactate testing. Further study of DFA a1 behavior in female participants, during constant load intervals, index stability over long periods of time and across diverse recording devices is recommended. If investigation into these matters remain consistent with the results presented here, obtaining a low intensity zone boundary by automated analysis of a training session *via* an appropriate wearable device may be possible.

## Data Availability Statement

The raw data supporting the conclusions of this article will be made available by the authors, without undue reservation.

## Ethics Statement

The studies involving human participants were reviewed and approved by University of Derby, United Kingdom (LSREC_1415_02). The patients/participants provided their written informed consent to participate in this study.

## Author Contributions

BR and TG conceived the study. DG and ND performed the physiologic testing. BR wrote the first draft of the article. BR and TG performed the data analysis. All authors revised it critically for important intellectual content, final approval of the version to be published, and accountability for all aspects of the work.

## Conflict of Interest

DG was employed by company Lattice Training. The remaining authors declare that the research was conducted in the absence of any commercial or financial relationships that could be construed as a potential conflict of interest.
